# Suppression of *Staphylococcus aureus* biofilm formation under a short-term impact of low-intensity direct current *in vitro* and in a rat model of implant-associated osteomyelitis

**DOI:** 10.22038/IJBMS.2023.72411.15938

**Published:** 2024

**Authors:** Evgenii Ovchinnikov, Tamara Silanteva, Maksim Stogov, Olga Diuriagina, Natalia Godovykh, Nadezhda Kubrak

**Affiliations:** 1 Experimental Laboratory, National Ilizarov Medical Research Centre for Traumatology and Ortopaedics, Kurgan, Russia; 2 Laboratory of Morphology, National Ilizarov Medical Research Centre for Traumatology and Ortopaedics, Kurgan, Russia; 3 Department of preclinical and laboratory research, National Ilizarov Medical Research Centre for Traumatology and Ortopaedics, Kurgan, Russia

**Keywords:** Biofilm, Direct current, Implant, Osteomyelitis, Rats, Staphylococcus aureus

## Abstract

**Objective(s):**

We investigated the effect of short-term low-intensity direct current (LIDC) on *Staphylococcus aureus*.

**Materials and Methods:**

The reference strain of *S. aureus* was used. Experiments were performed in agar culture and on a model of rat’s femur osteomyelitis. K-wires were used as electrodes. The exposure to LIDC of 150 μA continued for one minute. *In vitro* exposure was performed once. *In vivo* group 1 was a control group. Osteomyelitis was modeled in three groups but only groups 3 and 4 were exposed to LIDC four times: either from day 1 or from day 7 post-surgery. The effect was evaluated on day 21. Microbiological, histological, scanning electron, and light microscopy methods were used for evaluation of the LIDC effect.

**Results:**

Bacteria diameter, oblongness, and division increased 15 min after LIDC exposure in the culture around the cathode. After 24 hr, the amount of exomatrix was lower than in the control test, and the cell diameter and roundness increased. Similar changes around the anode were less pronounced. *In vivo*, biofilm formation on the intramedullary wire cathode was suppressed in group 3. In group 4, detachment and destruction of the biofilm were observed. The formation of *S. aureus* microcolonies was suppressed, and the adhesion of fibroblasts and immune cells was activated. LIDC did not stop the development of the osteomyelitis process.

**Conclusion:**

Short-term exposure to LIDC suppresses *S. aureus* biofilm formation on the implant cathode surface in the acute and early postoperative period but does not have an impact on the development of osteomyelitis.

## Introduction

The use of medical implants and percutaneous orthopedic fixators in trauma and orthopedic practice always carries a risk of hospital- and community-acquired infection. Implant-associated infection almost inevitably leads to the replacement of the implant or bone fixation pin (1-5). The results of numerous clinical studies show that *Staphylococcus aureus* has been recognized as the main cause of such opportunistic infections (6-10).

*S. aureus* frequently promotes the formation of a multilayer biofilm consisting of DNA, proteins, and an exopolysaccharide matrix that unites bacterial clusters (10-13). Bacterial colonization occurs more frequently and at a faster rate on hard and rough surfaces, therefore bone tissue and medical implants are ideal sites for bacterial adhesion as the first step in biofilm formation. The subsequent development of a polymeric extracellular matrix and aggregated multicellular communities, united by a “quorum sense”, decreases their sensitivity to antimicrobial therapy (14, 15).

Biofilm formation is associated with such life-threatening complications as implant-associated infection and chronic osteomyelitis (1, 2, 8). Biofilms play a key role in the development of chronic infectious processes since they increase the resistance of microorganisms to immunity factors and antibacterial agents by 100-1000 times in comparison with planktonic cell forms (7, 13, 14). The main strategies for the treatment of these complications are antibiotic therapy and surgical debridement (1, 2). Due to the problems of increased contamination of medical implants and resistance of microorganisms to antibiotics, there is a high demand for new effective technologies to fight them (15). One of the promising research directions is finding ways of preventing biofilm formation and dispersal seen as a potential treatment measure for persistent infections, including the ones caused* by S. aureus*. This approach is aimed at returning bacteria to a metabolically active planktonic state in order to increase their susceptibility to conventional antibiotics (7, 15, 16).

Over the past decades, the impact of electric current on biofilm-forming microorganisms has been actively studied in order to enhance the effectiveness of disinfectants and antibacterial drugs (13). Data on the impact of electric current that features varying characteristics have been constantly updated and resulted in new developments. For clinical use, modes of short-term exposure to low-intensity electric current seem most relevant since they ensure electrochemical and toxic safety for humans (3, 17, 18). However, their effect on *S. aureus *biofilm formation has been as yet insufficiently studied. Based on the results of our previous work (17), we assumed that the growth of the *S. aureus* bacterial biofilm might be suppressed under short-term exposure to low-intensity direct current (LIDC).

The purpose of our study was to investigate the impact of short-term exposure to LIDC on *S. aureus *biofilm formation in *in vitro* dense culture and in an *in vivo* model of murine implant-associated osteomyelitis. 

## Materials and Methods

***S. aureus culture and experimental model of murine femur osteomyelitis***

**Figure 1:**
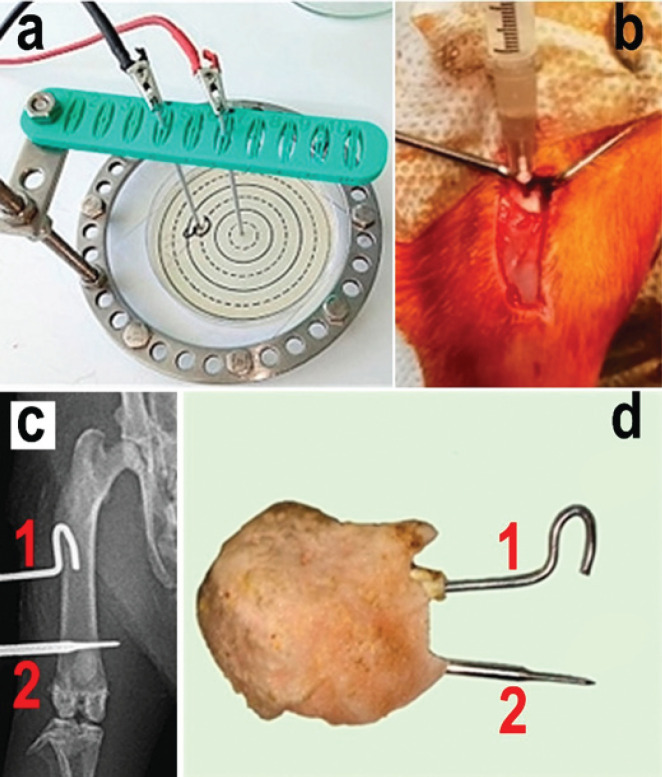
Experimental models for studying the impact of low-intensity direct current (LIDC) on *Staphylococcus aureus* biofilm formation

The reference strain of *S. aureus *(*subsp. aureus *ATCC 29213) was used to conduct the study. The test microorganism was cultivated on beef-peptone agar (NITsF Ltd, Russia) at 37 ^°^C for 18-24 hr. A standard microbial suspension was used for inoculation, equivalent to 0.5 according to the McFarland standard, obtained by direct suspension of colonies in a sterile isotonic solution. Fifty microliter of the prepared suspension of the tested strain was seeded on a Petri dish with Muller-Hinton medium (NITsF Ltd, Russia) (Figure 1a). The inoculum was introduced no later than 15 min from the moment of preparation. 

***LIDC device***

LIDC impact was produced with a Biopotentiometer BMP-02 medical device (NOP Kvant, Russia, registry #92/135-49). Its technical description and principles of action were previously described (17). The LIDC device operating mode was 150 μA, and the duration of exposure was one minute. This mode of electrical stimulation was chosen on the basis of our previous *in vitro* studies on the model of the broth culture of *S. aureus* (17). Two Kirshner wires (ᴓ 1 mm, stainless steel 12Х18Н9) were used as electrodes.

***LIDC impact on the S. aureus dense culture medium***

In the experiment *in vitro*, the wires were fixed in a stand made of dielectric material (Figure 1a). A positively charged electrode (anode) was placed in the center of a freshly prepared lawn of *S. aureus* microbial culture. A negatively charged electrode (cathode) was installed at a distance of 20 mm from the anode. LIDC exposure was carried out for one minute at 150 μA. No later than 15 min after the exposure, samples were taken from equidistant areas of the surface of the agar culture in the area of the anode and cathode with a microbiological 1-mm loop. The material was applied as a smear on the surface of a glass slide. Next, the cups were placed in a thermostat at a temperature of 37 °C for incubation for 24 hr. After the end of the incubation, smears from similar zones of the microbial culture were re-prepared for a comparative study. Six parallel tests were performed. For each test, the growth of culture without exposure to LIDC was monitored for control. 

***LIDC impact on the experimental model of murine femur osteomyelitis***

We used the experimental model of chronic osteomyelitis of the murine femur developed and reported in our previous work (19). All operations on animals were performed in the operating room under general anesthesia. Rometar solution 2% (2 mg/kg)(Bioveta, Czech Republic) was used for premedication, and Zoletil-100 solution (15 mg/kg)(Virbac Sante Animale, France) for anesthesia. The femoral diaphysis was reached through an open approach. The culture of the reference strain *S. aureus* ATCC 29213 at a concentration of 10^8 ^ml^-1 ^was injected into the diaphysis in a volume of 50 µl (Figure 1b). Two Kirshner wires (ᴓ 1 mm, stainless steel 12Х18Н9, state standard 5632 2014) were introduced in the rat’s femur and were intraosseous electrodes. The wires were cleaned with 70˚ medical alcohol and passed autoclaving. The intramedullary wire was bent and the transosseous wire was straight. The surgical wound was stitched with Vicryl 4/0 (Johnson & Johnson International, USA)**.** The wires were connected and fixed with a non-conducting self-hardening plastic material Protacryl-M (Soma Ltd, Ukraine)(Figure 1c, d).

The experiment *in vivo* was conducted on 24 Wistar rats of both sexes aged 11-12 months and having an average weight of 396±17.6 g. The total duration of the experiment was 21 days since the surgery. 

The animals were randomly divided into 4 groups, 6 animals in each group. In group 1, osteomyelitis was not modeled but wires were introduced. Osteomyelitis was modeled in the other three groups along with wire introduction. In groups 1 and 2, LIDC was not used. In group 3, exposure to LIDC was performed on the day of surgery and days 2, 4, and 6 after it. Group 4 rats were exposed to LIDC on days 7, 9, 11, and 13 after surgery. The cathode (-) was always connected to the intramedullary wire in the proximal third, and the anode (+) was always connected to the transosseous wire in the distal third of the femur (Figure 1c). 

Material for morphological study was taken on day 21 of the experiment.

Experimental animals were kept in individual cages and had free access to a standard diet and drinking water. The general state of the animals, their behavior, the condition of the tissues in the area of the wires, and the control of their limb function were assessed daily. Antimicrobial therapy in the postoperative period was not administered in order to avoid distortion of the study results. 

***Biofilm formation study with SEM***

The morphology of bacterial films in smears from the surface of a dense culture of *S. aureus *and on the surface of the wires after implantation was studied using a Zeiss EVO MA 18 scanning electron microscope with a secondary electron detector (Carl Zeiss Group, Germany). The samples were prepared according to a patented method: the material was fixed in a mixture of paraformaldehyde and glutaraldehyde solutions in phosphate buffer at pH 7.4 and a temperature of 4 ^°^C, then washed in phosphate buffer and distilled water. Next, the samples were dehydrated step by step in ethanol solutions and impregnated with camphene, followed by sublimation to preserve the native cell morphology, as described previously (20, 21). The dried samples were sputtered with platinum twice for 10 min in an IB-6 ion sprayer (JEOL, Japan) and examined in high vacuum at a voltage of 8-20 kV with a focal length of 5.5-6.5 mm. 

***Morphometry of Staphylococcus aureus cells***

The morphology of bacterial cells and intercellular substance was visually assessed on digital images. Morphometric measurements were performed using the VideoTesT-Master Morphology analyzer (Russia). A randomized sample for analysis in each test was 500 microbial cells. The obtained values were used to calculate the average diameter, roundness, and oblongness coefficients according to the following formulas:

Mean diameter=*D*_max_*+D*_min_*/2*, where *D*_max_-maximum and *Dmin*-minimum cell diameter; 

Roundness= 4A/ f^2^∙π, where *А*-area,* f*-Feret maximum diameter;

Oblongness *F*_y_ = D_max_/ D_min_, where *D*_max_- maximum, *Dmin*- minimum diameters of cells.

The proportion (%) of cells in the division stage (having a dividing septum but not divided yet) and the proportion (%) of solitary cells not included in multicellular groups were also evaluated. 

***Study of femur histostructure with light microscopy***

The extracted murine femurs were fixed in 4% neutral formalin, decalcified in the Richmann-Gelfand-Hill mixture, and dehydrated in increasing concentrations of alcohol. Paraffin-embedded bone blocks were microtomized using an HM 450 sliding microtome (Thermo Fisher Scientific, UK). Histological sections, 5-7 µm thick, were stained with hematoxylin and eosin. *S. aureus* in tissues was detected by Gram staining.

Automated digitization of histological preparations was performed in a PANNORAMIC Midi II BF slide scanner (3DHISTECH Ltd., Hungary) using the Whole-slide imaging technology. The study of digital histological preparations was performed using the PANNORAMIC Viewer software, version 2.4. 

***Pathomorphological identification of osteomyelitis types and stages***

Identification of osteomyelitis types was carried out in accordance with the classification of Grigorovsky (2013) (22). Jupiter score (23) and HOES (24) semi-quantitative scoring were used to objectify the histopathological signs of the osteomyelitis stage (Tables 1 and 2). 

***Statistical analysis***

Statistical processing and analysis of quantitative data were performed using the AtteStat program, version 12.1.7 (Rospatent Certificate No. 2002611109 dated June 28, 2002). The assessment of statistical hypotheses about the equality of the mean sample values was carried out using the parametric Student’s t-test or the non-parametric Mann-Whitney test after a preliminary checking for normality of distribution according to the Kolmogorov-Smirnov criteria. Intergroup differences were considered significant at *P*≤0.05.

***Ethical principles and regulatory standards***

Prior to the study, approval was obtained from the ethics board of our institution (min of the meeting No. 1 (71) dated April 28, 2022). All manipulations were carried out in accordance with the European Convention for the Protection of Vertebrate Animals used for Experimental and Other Scientific Purposes (Strasbourg, March 18, 1986).

## Results

***Suppression of S. aureus growth in a dense culture medium and biofilm formation in vitro***

The growth inhibition zone was less than 1 mm wide, both around the anode and around the cathode, when assessed visually 24 hr after seeding *S. aureus* on an agar lawn and exposed to LIDC of 150 μA for a min.

**Figure 2:**
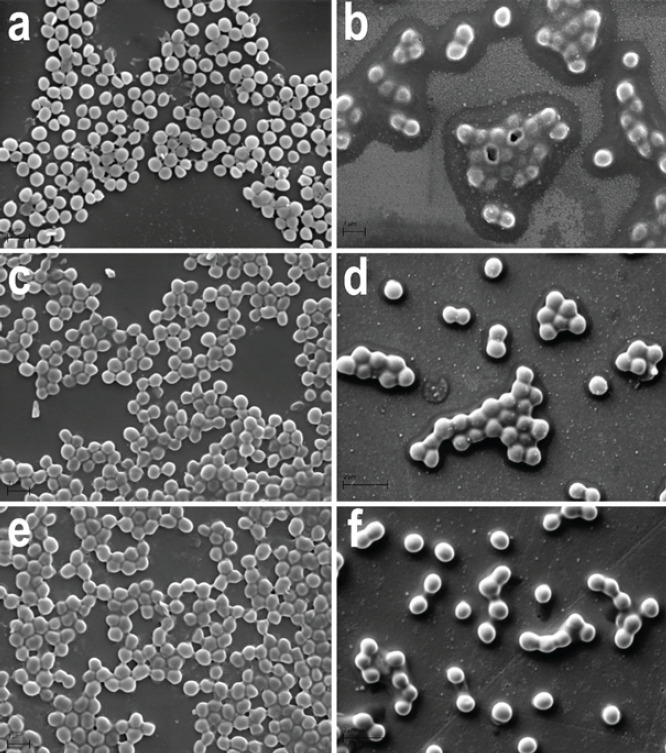
Impact of low-intensity direct current 150 µА for a minute on the *Staphylococcus aureus* morphology in a dense culture

In microbiological lawns without exposure (control) and 15 min after LIDC exposure, *S. aureus* bacteria did not have obvious morphological differences. They were rounded, slightly elongated, and located mainly in groups (Figure 2a, c, e).

The average cell diameter varied from 0.4 to 0.7 µm (Table 1). The exomatrix of bacterial cultures was sparse and had an amorphous consistency. Solitary deformed cells were observed both in the control and the LIDC tests. 

The average cell diameter around the cathode was significantly greater than in the anode region and the control tests 15 min after electric exposure. The bacteria were less round and more elongated. In the anode region, the values of the size and shape parameters did not differ from the data of the control group (Table 1).

The portion of dividing cells in the cathode region significantly exceeded the one in the control test and in the anode region. The portion of solitary cells, on the contrary, was significantly reduced. In the anode region, a statistically unconfirmed trend towards an increase in the number of dividing and a decrease in the number of solitary cells was also noted (Table 2).

After 24 hr of the experiment, the study of the shape and relief of the cell surface showed no signs of destructive changes in smears from the dense culture surface of *S. aureus*, both in the control test and in the LIDC test. Bacterial cells in all groups were larger than at the beginning of the experiment, their average diameter varied from 0.7 to 0.9 µm (Table 1). In the control test, there were morphological signs of biofilm formation. Abundant exocellular matrix was found on the surface of bacterial clusters and isolated cells, as well as in the spaces between them. It contained numerous spherical granules less than 0.1 µm in size. The same granules decorated the surface of most cells. Each group of bacteria or each solitary cell was surrounded by an inclusion-free zone of a homogeneous matrix, 0.3-0.8 µm wide (Figure 2 b).

In cultures subjected to LIDC, the least pronounced changes were observed in the anode region. As in the control test, fields of intercellular substance containing a granular component and a homogeneous matrix in the pericellular spaces were found there. However, the total amount of extracellular matrix and the content of dense granules in it were lower. Granules on the cell surface were also few (Figure 2d). 

In the area of the cathode, the amount of intercellular substance was minimal; it was concentrated mainly on the surface of cell groups and in a limited space around them. The granular component was scarce and absent on the cell surface. Zones of homogeneous matrix around cells and cell clusters were not observed (Figure 2f).

The average cell diameter in the cathode region was significantly higher; the bacteria were more rounded and less elongated, more often located solely than in the anode region and the control test. In the anode region, the same trends were noted, but the differences were not statistically significant in relation to the data of the control group (Table 1). Proliferating cells were found neither in the control nor in the LIDC tests. The highest proportion of solitary cells was detected in the area of the cathode. In the anode region, the increase in the proportion of solitary cells was not statistically significant in relation to the control (Table 2). 

***In vivo S. aureus biofilm formation 21 days after wire implantation***

**Figure 3:**
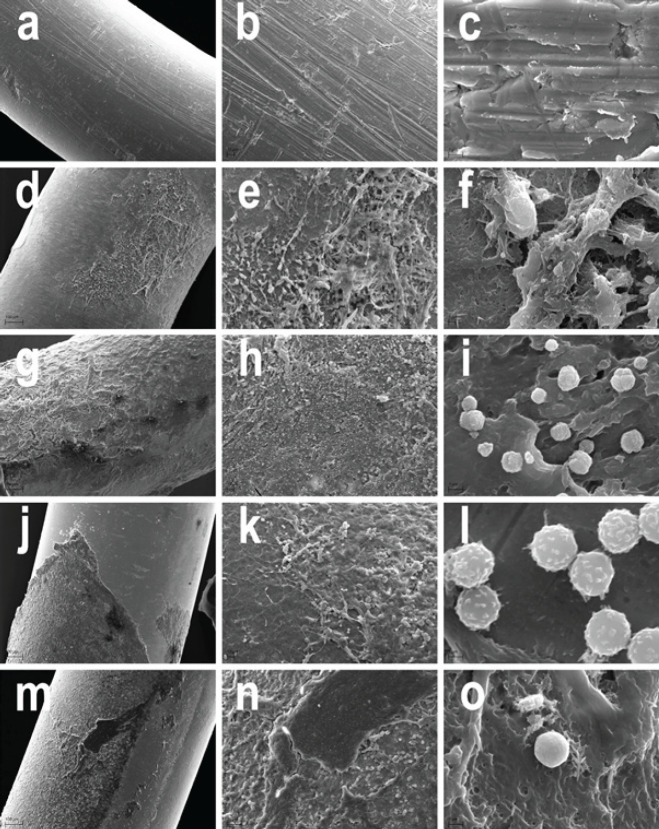
Microstructure of the surface of the electrode (cathode) steel wire

The formation of *S. aureus* biofilms on the surface of cathode wires was studied on a model of femoral osteomyelitis. The surface of the wires, similar to those used for clinical implantation, had the following microfeatures: roughness along its entire length, notches, depressions, and furrows that were from one to tens of micrometers long (Figure 3 a-c).

On the surface of the wires in control group 1, an organic matrix and numerous fibroblast-like cells with signs of active fibrillogenesis were found (Figure 3 d-f). In group 2, the surface of the extracted wires was covered with a mature *S. aureus* biofilm. The multilayer exomatrix of the biofilm adhered tightly to the surface of the implant and formed characteristic mushroom-shaped structures. It had channels and cracks typical of the initial stage of mature biofilm destruction. Numerous bacterial microcolonies were located on the surface of the exogenous matrix. Adherent leukocytes were not detected (Figure 3 g-i). Fragments of a thin biopolymer film without signs of bacterial adhesion were detected on the surface of the wire in group 3. It contained spreads of fibroblast-like cells, fibrillar structures of the extracellular matrix, and a large number of adherent leukocytes (Figure 3 j-l). In group 4, exfoliating fragments of a dense exomatrix with deep cracks, similar to a collapsing bacterial biofilm, were found on the implant surface. Signs of bacterial *S. aureus* microcolonies were not observed. Attached fibroblast-like cells, fibrillar matrix, and adherent immune cells were located on biofilm-free areas of the wire surface (Figure 3 m-o).

***Histological structure of murine femur 21 days after wire implantation***

**Figure 4:**
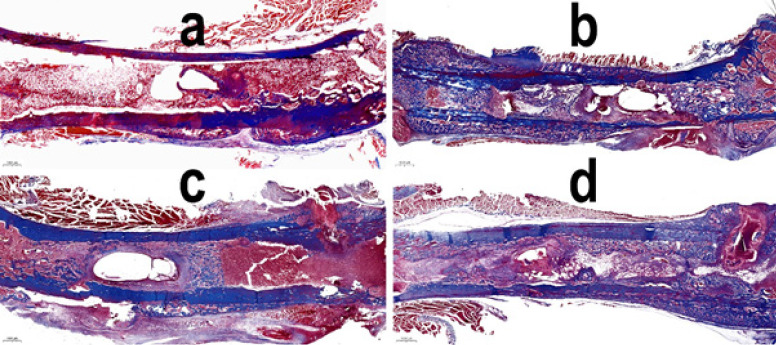
Histological structure of the rat femur metadyaphysis on experiment day 21

**Figure 5:**
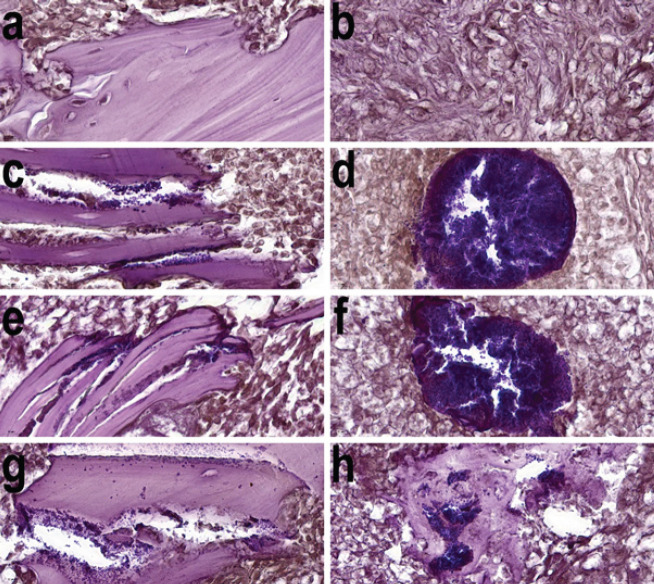
*Staphylococcus aureus* in the rat femur metadyaphysis on experiment day 21

The medullary cavity of the femur in group 1 rats was filled with red and yellow marrow with a rare trabecular meshwork. The wire canals were surrounded by a thin “envelope” of compacted coarse-fibered trabeculae; its inner walls were lined with granulation tissue. The compact plate of the femur had signs of osteoblastic-osteoclastic remodeling in the area where the wires were inserted, and small stratifications of cancellous bone substance were determined on its surface (Figure 4a). Inflammatory and bacterial cells were not detected (Figure 5 a, b).

A histological study of the femoral bone tissue in the area of osteomyelitis modeling and wire insertion in the groups both with and without electrical stimulation showed the same morphological picture that corresponded to chronic osteomyelitis (fibrosis with macro- and microabscess (Figure 4 b-d).

On the periosteal surface of the compact plate, there were layers of cancellous bone substance in the stage of active formation. The areas of compact bone adjacent to the osteomyelitis cavity underwent active osteoblastic and osteoclastic remodeling. In the Haversian canals of necrotic areas, there were colonies of staphylococci. Bone sequesters also colonized by *S. aureus* were visualized in the wire tracts.

In the medullary canal of the diaphysis, an osteomyelitis cavity containing detritus and *S. aureus* microcolonies was found. The involucrum of the osteomyelitis cavity was formed by a finely looped network of endosteally formed reticulofibrous bone trabeculae with reactively altered loose connective tissue into the intertrabecular spaces. The fibrous membrane of the cavity was infiltrated with inflammatory cells, mainly neutrophilic granulocytes; it contained infected bone sequesters and microabscesses with centrally located staphylococcal microcolonies (Figure 5 c-h).

***Pathomorphological identification of osteomyelitis types developed in the rat femur***

Semi-quantitative scoring of osteomyelitis stages using the Jupiter scale (23) showed that the threshold value of 15 points was exceeded in the groups of modeled osteomyelitis and it indicated that an acute course of chronic osteomyelitis developed. Similarly, the HOES score exceeded the threshold of 6 points defined for active chronic osteomyelitis (24). Statistical analysis of the results of assessing the activity of the osteomyelitis process did not reveal significant intergroup differences (Table 3).

## Discussion

Our electron microscopic study of the ultra-short LIDC impact on the *S. aureus* dense culture in the mode that has not been used earlier (150 μA for a minute) yielded a number of new findings. We found that despite the absence of suppression areas or signs of cell death, there were signs of morpho-functional alterations of the bacterial cells in the area of stainless steel cathode wire *in vitro*. The morphometric study revealed an increase in the average diameter, oblongness, and activation of cell proliferation 15 min after the LIDC exposure (Tables 1 and 2). Twenty-four hours after the exposure, we observed changes in the shape and size of the cells, as well as suppression of formation and alteration in morphological characteristics of the exomatrix which plays a leading role in biofilm formation. The changes of a similar character in the area of the anode were weakly expressed and were not statistically significant (Figure 2 a-f; Tables 1, 2).

In the* in vivo* model of implant-associated osteomyelitis of the rat’s femur, the suppression of biofilm formation was revealed on the surface of the intramedullary cathode wire in the LIDC mode used on the operation day and on post-surgery days 2, 4, and 6. Moreover, detachment and destruction of the biofilm were observed if the same mode was used on post-surgery days 7, 9, 11, and 13. Additional effects were suppression of *S. aureus* microcolony formation and activation of adhesion of cell inflammatory elements on the implant surface. It should be noted that such effects were prolonged as they were seen on post-surgery day 21 (Figure 3 a-o).

Our findings on the morphology of *S. aureus* cells and biofilms are consistent with the results of several previous studies. Atomic force microscopy (AFM) was used by Boudjemaa *et al*. to study the temporal evolution of the surface nanotopography and mechanical properties of *S. aureus* starting from bacterial adhesion to the first stage of biofilm formation for 24 hr. (25). The authors discovered an active formation of dense globular clusters of the exocellular matrix on the surface of adherent bacterial cells, which then accumulated in the intercellular space. Starting from 20 hr of cultivation, the cells abundantly secreted a soft polymeric substance, presumably containing polysaccharides. Their data correlate with the results of our study that showed similar abundant formation of globe-shaped conglomerates and amorphous polymer substances 24 hr after culturing on the agar lawn in the control group (Figure 2 a, b).

Under the impact of cathodic electrical stimulation in our study, the phenotype (shape, size) and biosynthetic activity of bacterial cells changed, affecting the formation of the exocellular matrix (Figure 2 a-f; Tables 1 and 2). Such phenotypic heterogeneity is consistent with the current microbiological paradigm, which considers colonies of genetically identical microorganisms as biosocial systems consisting of several heterogeneous clonal clusters of cells (bacterial phenotypes) that prevail and depend on changes in the environment (26).

Monteiro *et al*. established that *S. aureus* cells are globe-shaped before division and obtain oblongness in the last stage of the cell cycle, before division into two daughter cells by a previously formed septum (27). At the same time, the replication process of *S. aureus* is quite fast and takes about an hour on average (28). We observed a similar change in the shape and an increase in the number of cells in the division stage, Fifteen minutes after short-term LIDC exposure (Tables 1, 2). Presumably, the 150 μA LIDC regime for 1 min stimulated the proliferation of bacterial cells and did not act on them bacteriostatically. A similar result of exposure to electric current, determined by the method of counting log_10_ CFU/g of bone, was obtained by Schmidt-Malan *et al*. on experimental models of implant-associated osteomyelitis in rats after 21 days of continuous electrical stimulation with a constant electric current of 200 μA using platinum electrodes. Under those conditions, an increase in the number of *S. aureus* bacteria in the bones was observed compared with the control, but it was not statistically significant (13).

There is evidence that the negative charge of the surface of implant materials reduces the ability of *S. aureus* to attach due to interaction with similarly charged teichoic acids on the surface of microorganisms **(**15**)**, and also provokes the detachment of mature bacterial biofilms (5, 18). We observed the same effect in an *in vivo* experiment on a clinically relevant model of an “early” implant-associated infection on the surface of an intramedullary implanted cathode wire after electrical stimulation sessions (Figure 3 g-o). However, in most previous experiments, the assessment of bacterial contamination on the material surface was carried out immediately or shortly after the cessation of electrical exposure (3, 13, 18, 29-38). In our study, the morphological evaluation was delayed and showed that the anti-biofilm effect persisted for a long period after the termination of the LIDC sessions.

The researchers used various model systems *in vitro*: cultivation in liquid (17) and dense media (30, 35), on the surface of substrates made of various materials (3, 18, 31, 33, 34, 35-38) and medical devices (29; 33); and *in vivo*: implant-associated intraosseous inoculation (13) and intraosseous implantation of a biofilm on the surface of a titanium rod (37). The amplitude of electric current in selected studies ranged from 4 μA (29) to 10 mA (38). The duration of the electric exposure also varied, from 60 sec (17) to 21 days (13). A bacteriostatic effect was achieved with application of microampere currents *in vitro* in all experiments that were expressed in a decrease in the number and/or viability of bacteria, partial exfoliation of the biofilm, and was directly dependent on the current strength and duration of exposure (3, 13, 17, 18, 29-36). The bactericidal (electrocidal) effect, expressed in a decrease in the indicators by more than 90% compared with the control, was confirmed in several experiments with prolonged exposure to microampere electric current (18, 29, 33) and by applying a milliampere current (1 mA and 10 mA up to 1 hr) (37, 38). 

As *S. aureus *is a more frequent cause of biofilm formation in implant-associated infections than other microorganisms, the results obtained have a potential clinical relevance. In the *in vivo* model of osteomyelitis, the methods of control did not show bacteriostatic and bactericidal action of microampere current by applying continuous electrical stimulation for 21 days (13). The LIDC mode described by us also did not arrest the osteomyelitis in the murine femur (Figures 4 a-d and 5 a-h; Table 3). Thus, it cannot be used as an independent treatment method for implant-associated infection. However, a milliampere current of 1 mA effectively reduced the number of biofilm-forming bacteria on the implant surface and in the bone tissue within 1 hr of electrical exposure immediately after implantation (37).

Although it is difficult to compare the results due to the variety of materials used as electrodes and substrates for culturing microorganisms, as well as other model conditions, they confirm the effectiveness of LIDC stimulation in reducing the bacterial load on the surfaces of medical implants and in their immediate vicinity. However, as shown in a number of studies, not all modes of electrical stimulation are electrochemically safe. Corrosion of electrodes during LIDC stimulation with a current strength of 300, 450, 500, 1060, and 1450 μA was proven (17; 35; 37). The use of weaker LIDCs, for example, 150 μA, does not lead to corrosion of electrodes during short-term exposure (17; 35; 37). At the same time, it is capable of exerting a bactericidal effect under certain model conditions, which the authors attribute to the generation of H_2_O_2_ at the cathode and chlorine at the anode (30). The use of this technology for suppressing *S. aureus* biofilm formation on the surface of implants and orthopedic fixators is consistent with proven methods of electrical stimulation for bone healing and skin wounds, which are effective if LIDC is applied (39-41).

The limitation of our study is that only one strain of *S. aureus* was tested. There are a variety of experimental conditions that should be tested in the future such as other implant materials and electrical exposure modes, and the effect of electrical impact on biofilm formation on the surface of the implants with an osteoinducing (calcium phosphate) coating. The question of the interaction of short-term LIDC exposure with various antibiotic therapy regimens remained outside the scope of our study. LIDC exposure radius was limited in the culture, so the effect of LIDC on the infection of the surrounding bone has not been established. The data obtained are of potential clinical interest for the prevention of implant-associated infection that occurs with the application of orthopedic transosseous metal elements (screws, wires) used in external fixation.

*In vitro*: The electrodes immersed in the microbiological lawn are fixed in a stand, the anode is in the center, and the cathode is on the periphery (a). *In vivo*: suspension of *S. aureus* was inoculated into the bone marrow cavity of rat femur (b); intramedullary (1) cathode proximally and transosseous (2) anode distally (c, d); both electrodes are connected by non-conductive self-hardening plastic (d)

**Table 1:** Low-intensity direct current (LIDC) impact on the *Staphylococcus aureus* cell size and shape in dense medium

Term	15 min after seeding						24 hr after seeding					
Parameter	D_mean_ (μm)		F_o_		F_у_		D_mean_ (μm)		F_o_		F_у_	
Value	M	SD	M	SD	M	SD	M	SD	M	SD	M	SD
Control	0.57	0.04	0.91	0.05	1.11	0.07	0.78^1^	0.079	0.88^1^	0.06	1.15^1^	0.09
Anode	0.58	0.04	0.91	0.04	1.11	0.06	0.79^1^	0.076	0.89^1^	0.05	1.13 ^1^	0.08
Cathode	0.62	0.06	0.88	0.05	1.16	0.08	0.82 ^1^	0.062	0.91 ^1^	0.04	1.11 ^1^	0.06

D_mean_–mean diameter, F_o_–roundness factor, F_у_–elongation factor; differences between the control and test samples in the same period of the experiment are significant at *p*_t_*≤*0.05 (underlined); 1-differences between eponymous samples at different periods of the experiment are significant at *p*_t_*≤*0.05

**Table 2:** Low-intensity direct current (LIDC) impact on the portion (%) of proliferating *Staphylococcus aureus* and solitary cells in dense medium

Term	15 min after seeding				24 hr after seeding			
Parameter	Proliferating cells		Solitary cells		Proliferating cells		Solitary cells	
Value	Ме	Q25-Q75	Ме	Q25-Q75	Ме	Q25-Q75	Ме	Q25-Q75
Control	2.3	1.8-6.5	3.1	2.0-3.9	0^1^	0-0	5.7^1^	3.4-7.9
Anode	3.1	2.0-4.0	2.0	1.9-2.0	0^1^	0-0	8.3^1^	7.0-9.1
Cathode	9.1	7.2-13.3	0.5	0.0-1.1	0^1^	0-0	16.3 ^1^	14.6-17.9-

Me is the median; Q25-Q75 - range between the 1st and 3rd quartiles; differences between the control and test samples in the same period of the experiment are significant at *p*_u_*≤*0.05 (underlined); 1 - differences between eponymous samples at different periods of the experiment are significant at *p*_u_*≤*0.05

Without low-intensity direct current (LIDC) impact (a, b); in the anode area (c, d); in the cathode area (e, f). Time after seeding: 15 min (left column) and 24 hr (right column). Electron microscopy scans. Magnification ×20000

Microroughness of the wire surface before implantation (a-c). Wire surface after 21 days of the experiment: attached fibroblast-like cells and intercellular matrix (d-f) on wires of the control group; mature biofilm of *Staphylococcus aureus* with complex morphology, spreading staphylococci microcolonies on wires of group 2 without low-intensity direct current (LIDC) exposure (g-i); a thin layer of an organic substrate with signs of fibrillogenesis and adherent leukocytes after LIDC exposure from experiment days 1 to 6 in group 3 (j-l); exfoliating fragments of a multilayer biofilm without spreading S. aureus microcolonies, signs of fibrillogenesis and adherent leukocytes in LIDC exposure from days 7 to 13 in group 4 (m-o). Representative electronic scans. Magnification: 250 (left), 1000 (center), 20000 (right)

Control group: periosteal layers of spongy bone in the area of the wires, newly formed bone trabeculae around the wire tract, red and yellow bone marrow in the intramedullary cavity (a); a complex of signs of a fibrous-abscessing form of osteomyelitis in group 2 without low-intensity direct current (LIDC) exposure (b), group 3 with LIDC exposure from days 0 to 6 after surgery (c); group 4 with LIDC exposure from day 7 to 13 after surgery (d). Paraffin histotopographic sections. Trichrome stain according to Masson. Digital lens ×1

In the control group, bacterial cells were not detected (a, b). Gram-positive microcolonies of *S. aureus* were detected in bone sequesters (c, e, g) and microabscesses of the fibrous membrane of the osteomyelitis cavity (d, f, h) in group 2 (c, d), in group 3 after low-intensity direct current (LIDC) exposure from days 1 to 6 (e, f), and group 4 from day 7 to 14 after surgery (g, h). Paraffin histological sections. Gram stain. Digital vol. ×100

**Table 3:** Histological osteomyelitis evaluation according to Jupiter (23) and HOES (24) semi-quantitative scores

Group	Jupiter*		HOES*	
	Ме	Q25-Q75	Ме	Q25-Q75
Control group 1	1	0-3	1	0-3
Test group 2	27	27-28	9	7-9
Test group 3	27	27-27	7	7-7
Test group 4	27,5	27-28	9	8.5-9.5

Me is the median; Q25-Q75 is the range between the 1st and 3rd quartiles; underlined: intergroup differences between the control and experimental samples are significant at *p*_u_*≤*0.05

## Conclusion

The short-term exposure to LIDC suppresses *S. aureus* biofilm formation on the implant cathode surface in the acute and early postoperative period but does not have an impact on the development of osteomyelitis. The data obtained are of potential clinical interest for the treatment of implant-associated infections.

## Authors’ Contributions

E O, T S, M S, and O D designed the experiments; E O, T S, M S, O D, N G, and N K performed experiments and collected data. E O, T S, M S, and O D supervised the research. T S, O D, N G, and N K analyzed the data. T S prepared the original draft. E O, M S, O D, and N G helped with writing and editing.

## Source of funding

This study was carried out within the framework of the State Research Assignment Program for 2021-2023: Personalization of methods for diagnosis and treatment of patients with osteoarticular pathologies complicated by purulent infection with the purpose of infection suppression and orthopedic recovery.

## Conflicts of Interest

The authors of this study do not have any conflicts of interest to declare.
